# Risk of *Pneumocystis jirovecii* Pneumonia among Solid Organ Transplant Recipients: A Population-Based Study

**DOI:** 10.3390/jof9010023

**Published:** 2022-12-22

**Authors:** Yih-Dih Cheng, Ching-Hua Huang, Shuo-Yan Gau, Ning-Jen Chung, Shiang-Wen Huang, Cheng-Yang Huang, Chien-Ying Lee

**Affiliations:** 1 School of Pharmacy, China Medical University, Taichung 40402, Taiwan; 2 Department of Pharmacy, China Medical University Hospital, Taichung 40402, Taiwan; 3 Department of Pharmacology, Chung Shan Medical University, Taichung 40201, Taiwan; 4 Department of Pharmacy, Chung Shan Medical University Hospital, Taichung 40201, Taiwan; 5 Institute of Medicine, Chung Shan Medical University, Taichung 40201, Taiwan; 6 School of Medicine, Chung Shan Medical University, Taichung 40201, Taiwan

**Keywords:** *Pneumocystis jirovecii* pneumonia, solid organ transplant, adjusted odds ratio

## Abstract

Few studies have comprehensively investigated the occurrence of *Pneumocystis jirovecii* pneumonia (PJP) among solid organ transplant (SOT) recipients. This study investigated the risk of PJP after organ transplantation. Each patient who underwent SOT was propensity-score-matched with four non-SOT individuals in terms of sex, age, insured salary, urbanization of residence, comorbidities, and year of enrollment. When considering the 3-year follow-up, the patients who had undergone SOT were at higher risk of PJP, with the adjusted odds ratio (aOR) being 17.18 (95% confidence interval (CI): 8.80–33.53). Furthermore, SOT recipients were also at higher PJP risk than the patients without SOT at 6 months, 1 year, and 2 years, with the aOR being 22.64 (95% CI: 7.53–68.11), 26.19 (95% CI: 9.89–69.37), and 23.06 (95% CI: 10.23–51.97), respectively. Patients comorbid with HIV infection, hematological malignancies, or vasculitis were at higher risk (aOR = 59.08, 95% CI = 20.30–171.92), (aOR = 11.94, 95% CI = 5.36–26.61), and (aOR = 21.72, 95% CI = 2.41–195.81), respectively. The recipients of SOT were at higher risk of PJP, and PJP can develop at any stage after transplantation. SOT recipients comorbid with HIV, hematologic malignancies, or vasculitis were at higher PJP risk.

## 1. Introduction

Patients who undergo solid organ transplant (SOT) are susceptible to opportunistic infections, which are a major cause of morbidity and mortality [1]. *Pneumocystis jirovecii* (PJ) is a globally prevalent opportunistic fungus that can cause infection in patients who undergo SOT. Immunocompetent hosts can clear infections without evident clinical consequences, whereas immunocompromised hosts are more likely to develop disease as a consequence of possible reinfection and reactivation of latent infection [2]. Studies have reported the transmission of PJ through the respiratory route in both immunocompetent and immunocompromised animals, and PJ is highly likely to spread among humans through human-to-human transmission [2].

PJ can localize in the alveoli of human lungs and cause pneumonia [3]. *Pneumocystis jirovecii* pneumonia (PJP), formerly known as *Pneumocystis carinii* pneumonia, is an opportunistic fungal infection that most commonly affects immunocompromised individuals [4].

Because studies on the risk of PJP among SOT recipients are scarce, epidemiological studies should be conducted to investigate the risk of PJP in this group, especially those using nationwide databases. The present study investigated the risk of PJP after organ transplant between 2001 and 2018 by using data from the National Health Insurance Research Database (NHIRD) of Taiwan.

## 2. Materials and Methods

### 2.1. Data Source

This study performed a secondary analysis of patient data in the NHIRD collected over the period 2001–2018 and published by the Health and Welfare Data Science Center (HWDC), Ministry of Health and Welfare, Taiwan. The NHIRD contains details of beneficiaries enrolled in Taiwan’s National Health Insurance (NHI) program, NHI enrollment files, and medical service data (e.g., diagnoses, prescription drugs, and examinations). The NHI program is a compulsory single-payer health-care program that provides comprehensive health care for >99% of the residents of Taiwan. Diagnostic data from the period before 2016 are coded in accordance with the International Classification of Diseases, Ninth Revision, Clinical Modification (ICD-9-CM), whereas data from the period after 2016 are coded in accordance with the tenth revision (ICD-10-CM).

### 2.2. Ethical Approval

This study was conducted in accordance with the Declaration of Helsinki. The data used in the analysis were released by the HWDC and had scrambled random identification numbers for insured patients to protect the privacy of the beneficiaries. The database is anonymous, and the HWDC deidentified the insured patients to protect their privacy. The requirement for informed consent was thus waived. This study protocol was approved as completely ethical by the Institutional Review Board of Chung Shan Medical University Hospital, Taiwan (No: CS2-21134).

### 2.3. Study Participants

Patients who received SOT between 2002 and 2015 were enrolled in the study, including those who underwent renal (ICD-9-CM V42.0), liver (ICD-9-CM V42.7), heart (ICD-9-CM V37.51), or lung (ICD-9-CM V42.6) transplant. Patients who had received more than one SOT, had received a diagnosis of related infectious disease before SOT (e.g., PJP, cytomegalovirus disease, and herpes simplex virus), or for whom there was insufficient information regarding the study variables were excluded. Propensity score matching (PSM) was used to obtain 1:4 matching for each patient receiving SOT. The characteristics selected for PSM were sex, age, insured salary, urbanization of residence, Charlson comorbidity index (CCI), and year of inclusion in the study.

**Figure 1:**
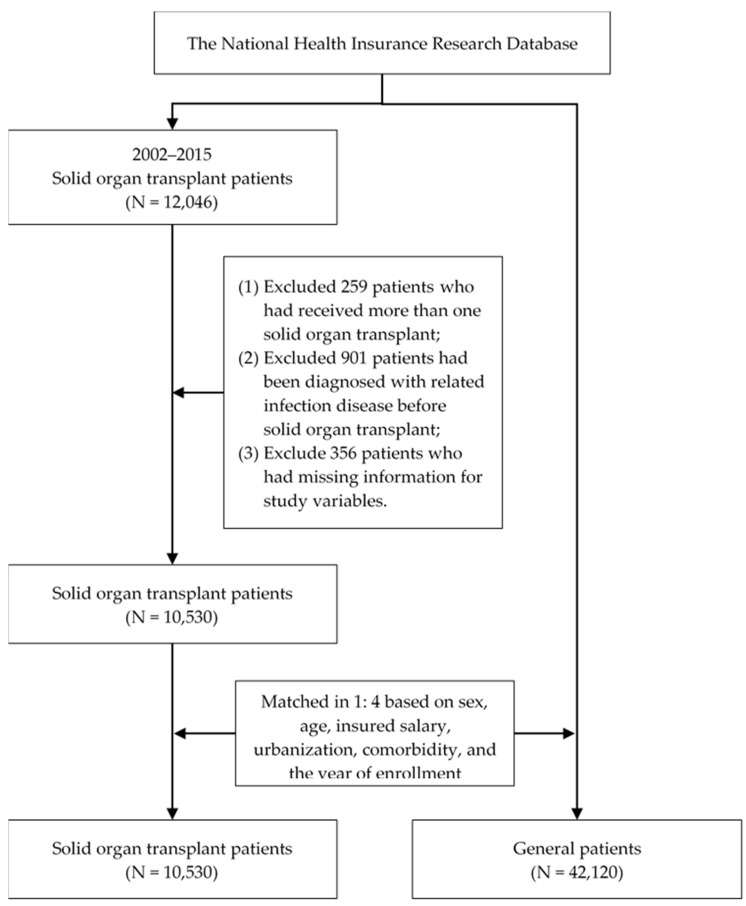
The flow of participant selection.

After matching, the data of 10,530 patients who received SOT (6179 kidney transplants, 4281 liver transplants, and 70 lung transplants) and 42,120 general patients were included in the study. Figure 1 illustrates the flow of participant selection for the study.

### 2.4. Study Design

The present study had a cross-sectional design, employed the NHIRD of Taiwan, and had a 3-year follow-up period to assess the risk of PJP among SOT recipients. The incidence of PJP infection was identified in accordance with the ICD-9-CM code 136.3 or ICD-10-CM code B59. The observation date was started on the date of patients receiving the SOT. The control variable contained sex, age, socioeconomic status (insured salary, urbanization of residence), comorbidities, and medication (trimethoprim–sulfamethoxazole (TMP–SMX), corticosteroid). Comorbidities were identified on the basis of the data of outpatient department visits and in the hospital admissions database for the period 2 years preceding the observation date. The comorbidities considered were hypertension (ICD-9-CM 401–405), hyperlipidemia (ICD-9-CM 272.0–272.4), hepatitis C (ICD-9-CM 070.70 and 070.4–070.5), chronic kidney disease (CKD; ICD-9-CM 585), chronic obstructive pulmonary disease (COPD; ICD-9-CM 490–492 and 494–496), HIV (ICD-9-CM 042–044), requirement of dialysis, rheumatoid arthritis (RA; ICD-9-CM 714), inflammatory bowel disease (IBD; ICD-9-CM 555, 556), systematic lupus erythematosus (SLE; ICD-9-CM 710.0), psoriasis (ICD-9-CM 696), Sjogren syndrome (ICD-9-CM 710.2), hematological malignancies (ICD-9-CM 200-209), and vasculitis (ICD-9-CM 709.1). The patients who received dialysis were categorized in accordance with whether they received peritoneal dialysis or hemodialysis during their outpatient department visits and in accordance with the hospital admission database. Medication was defined by the anatomical therapeutic chemical (ATC) code that the corticosteroid was A07EA03, H02AB06, H02AB07, and S02BA03, and the TMP–SMX was J01EE01.

### 2.5. Statistical Analyses

All statistical analyses in the study were performed using SAS software version 9.4 (SAS Institute, Cary, NC, USA). PSM was used to obtain the comparison. PSM is a statistical matching technique that can be used to reduce potential confounding caused by unbalanced covariates in nonexperimental settings. A propensity score is a probability that is calculated using a logistic regression model. The score is a unit of a certain characteristic assigned to a patient who received SOT. These scores can help reduce or eliminate selection bias in observational studies by accounting for the characteristics of control individuals. After the study participant selection, the chi-squared test was used to evaluate the distribution of baseline characteristics between the SOT and non-SOT groups. We investigated the risk of PJP among the SOT recipients through multiple logistic regression analysis with adjusted relevant variables, and the results are presented as adjusted odds ratios (aOR) with 95% confidence intervals (CIs). Furthermore, we conducted sensitivity analysis to investigate the risk of PJP among SOT recipients with different cohorts and at different follow-up periods. The different cohorts were patients with CKD and HIV, respectively. The different follow-up periods were the 6-month, 1-year, and 2-year follow-ups. All *p*-values < 0.05 were considered statistically significant.

## 3. Results

Table 1 lists the basic characteristics of SOT recipients and general patients with PJP after PSM. The data of 52,650 participants were used in the study. The average age of the SOT recipients was 47.40 ± 14.31 years, whereas that of the general patients was 49.07 ± 15.29 years. As expected, the characteristics of the matching variables—including sex, age, insured salary, urbanization of residence, and CCI—were similar between the SOT recipients and general patients (*p* > 0.05). Among SOT recipients, 4698 (47.18%) had hypertension, 1621 (15.39%) had hyperlipidemia, 1220 (11.59%) had hepatitis C, 5331 (50.36%) had CKD, 5104 (48.47%) required dialysis, and 478 (4.54%) had COPD. Furthermore, the distribution of each comorbidity exhibited a significant difference between the SOT recipients and general patients (*p* < 0.001). Among the SOT recipients, 5618 (53.35%) had used the TMP–SMX, and 7292 (69.25%) had used the corticosteroid. The proportion of those using TMP–SMX and corticosteroids among the SOT recipients was significantly higher than that of the general patients (*p* < 0.001).

The incidence rate of PJP in SOT recipients is shown in Table S1 in the Supplementary Materials. As displayed in Table 2, with other relevant influencing factors controlled for, the risk of developing PJP was higher among the SOT recipients, with the aOR being 17.18 (95% CI: 8.80–33.53), than among the general patients. In the subgroup analysis model, patients with lung transplants had the highest risk of PJP (aOR = 62.33, 95% CI = 16.95–229.22). Patients with liver transplants (aOR = 16.82, 95% CI = 8.11–34.89) and patients with kidney transplants (aOR = 15.03, 95% CI = 6.37–35.44) were at the second-highest risk level. Patients aged 41–50 or ≥61 years were at higher risk of developing PJP compared with those aged below 40 years (aOR = 1.75, 95% CI = 1.09–2.81; aOR = 2.01, 95% CI = 1.14–3.53, respectively). The PJP risk of the patients with hypertension was not higher than that of those without hypertension (aOR = 1.32, 95% CI = 0.89–1.96); however, patients with HIV infection were at higher risk (aOR = 59.08, 95% CI = 20.30–171.92), patients with hematological malignancies were at higher risk (aOR = 11.94, 95% CI = 5.36–26.61), and patients with vasculitis were at higher risk (aOR = 21.72, 95% CI = 2.41–195.81). Patients receiving TMP–SMX and corticosteroids both had a higher PJP risk, but there was no statistically significant difference.

Table 3 displays the sensitivity analysis of PJP incidence in the different observation periods. With other relevant influencing factors controlled for, the risk of developing PJP was higher among the SOT recipients than among the general patients, with the aOR being 22.64 (95% CI: 7.53–68.11), 26.19 (95% CI: 9.89–69.37), and 23.06 (95% CI: 10.23–51.97) at the 6-month, 1-year, and 2-year follow-ups, respectively. In terms of comparison with CKD patients, the PJP risk was higher among the SOT recipients, with the aOR being 17.85 (95% CI: 6.53–48.81), 13.05 (95% CI: 6.18–27.56), 9.85 (95% CI: 5.25–18.48), and 10.19 (95% CI: 5.68-18.27) at the 6-month, 1-year, 2-year, and 3-year follow-ups, respectively. In terms of comparison with HIV patients, the PJP risk was lower among the SOT recipients, with the aOR being 0.57 (95% CI: 0.56–0.57), 0.28 (95% CI: 0.27–0.28), 0.24 (95% CI: 0.23–0.24), and 0.23 (95% CI: 0.16–0.33) at the 6-month, 1-year, 2-year, and 3-year follow-ups, respectively.

## 4. Discussion

Few large-scale epidemiological studies have evaluated the risk of PJP among SOT recipients. In the present large population-based study, PJP was discovered to be able to develop at any post-transplant stage. After 6 months, 1 year, 2 years, and 3 years of follow-ups, the patients who underwent SOT were at higher risk of PJP, and the risk was found to be highest after 1 year after the transplant. Lung transplant recipients had the highest PJP risk, followed by the liver and kidney transplant recipients. Moreover, patients aged 41–50 or >60 years were at higher risk of developing PJP than younger patients. In addition, patients with comorbid HIV were at higher risk of developing PJP.

The common symptoms of PJP include low-grade fever, cough, and shortness of breath [5]. Serum β-1, 3-D-glucan analysis and quantitative real-time polymerase chain reaction (qPCR) have become increasingly valuable diagnostic tools. A French study proposed a strategy for a convenient diagnostic performance of PJP infection: Serum β-1, 3-D-glucan is helpful as a marker for the diagnosis of PJP and also demonstrated that qPCR in nasopharyngeal aspirate had higher sensitivity and specificity in diagnosing PJP [6]. Diagnoses of PJP have increased among HIV-negative patients, particularly among patients receiving immunosuppressive treatment for hematological malignancies, solid tumors, or SOT [7]. The development of PJP in SOT recipients has been linked to specific immunosuppressive drugs [8]. PJ can cause outbreaks among SOT recipients who are administered immunosuppressive treatment [9].

The progression of PJ infection in the human immune system is complex [10]. The adhesion of PJ triggers an immune response of the host, thereby causing severe lung injury in immunocompromised patients. The host immune response against PJP involves complex interactions between CD4+ T cells, CD8+ T cells, alveolar macrophages, neutrophils, and soluble mediators (including leukotrienes, prostaglandins, and histamine) that can facilitate the clearance of infection [11]. Low CD4+ T cell, CD8+ T cell, and NK cell counts were associated with poorer PJP prognosis among HIV-negative individuals [10].

Historically, trimethoprim–sulfamethoxazole (TMP–SMX) is a standard drug for the prophylaxis of PJP in immunosuppressed patients [12]. In the absence of routine implementation of standard PJP prophylaxis among SOT individuals, the overall incidence was approximately 5% to 15% [13,14]. The widespread use of TMP–SMX for therapy and prophylaxis of PJP has led to the concern that sulfa resistance could develop in PJ [15]. However, this reduced susceptibility is not always equal to PJ becoming fully resistant to these drugs [16,17]. Atovaquone is an alternative agent for oral use that can be used in the treatment in mild to moderately severe cases [18].

This study revealed that SOT recipients were at higher risk of PJP over 6-month, 1-year, 2-year, and 3 year follow-up periods. PJP may develop at any stage after transplantation. PJP among SOT recipients is a critical issue related to the transplantation of solid organs [19]. One study reported that PJP occurs in approximately 1% of SOT recipients [20]. Nosocomial infections have been identified in renal, liver, and heart transplant recipients [21,22]; these infections occurred through human-to-human transmission in the hospital environment and had incubation periods of up to 150 days [23]. PJP has become a prevalent respiratory opportunistic infection in severe immunocompromised transplant recipients [24]. Notably, CD4+ lymphocyte count <200 cells/μL is the primary risk factor for PJP presentation in these immunocompromised patients [25]. Additionally, recent analyses of available metagenomic data sets have focused on the frequent air shedding of *Pneumocystis jirovecii* and it transmission from PJP-confirmed patients, supporting early epidemiologic analysis data [26].

Incidence of PJP is variable according to the type of SOT. Our study demonstrated that lung transplant recipients were at highest risk for PJP development, followed by the liver and kidney transplant recipients. PJP infection rate depends on the type of transplantation and is greater in heart, lung, and combined heart–lung transplant recipients than in kidney or liver transplant recipients, regardless of whether these recipients receive prophylaxis or not [8]. Several early studies report that *Pneumocystis jirovecii* infects 0.3%–2.6% of SOT recipients [27,28,29]. A UK study in 2017 estimated the incidence of PJP among SOT recipients to be 5.8%, 5.5%, 1.2%, and 0.3% for lung/heart and lung, heart, liver, and kidney transplants, respectively [30]. Lung transplantation is an identified risk factor for PJP. A French study showed that the annual incidence rate of PJP was 2.7/1000 patients/year among lung transplantation recipients [31]. PJP has an incidence about 2–10% in heart transplant recipients without prophylaxis [32]. PJP has a relatively high incidence of 1%-11% among liver transplant recipients without prophylactic treatment [33]. The number of PJP cases increased 388% from 2006 to 2010 at an average annual increase of 9% in England, while the number of kidney transplant recipients increased by only 25% [34].

PJP is a severe and potentially fatal opportunistic infection that occurs in immunosuppressed patients. We discovered that SOT recipients were at the highest risk of developing PJP over 1 year after the transplantation and that they remained at high risk even after 2 and 3 years. Life-threatening infections are more likely to occur within the first 6 months after transplantation; these infections are related to the use of high-dose immunosuppressive drugs during this period [35,36]. During the period of peak immunosuppression, infections typically occur later (post-transplantation months 1 to 12), and the majority of infections are opportunistic infections [1,37]. The incidence of PJP among SOT recipients was high during the first year after the transplantation [38]. The risk of PJP in SOT recipients was considered to be highest in the 12 months after transplantation. In the absence of prophylaxis, PJP occurs in 5–15% of transplantation recipients, with the mortality rate being up to 50% depending on the organ that was transplanted [29,39]. However, the global prevalence of PJP among SOT recipients during the second year after transplantation is relatively low (<0.6%) [29]. After the second year, the risk of developing PJP is low and persists with time, but some long-term risks may remain for a long period [29,40].

This study demonstrated that SOT recipients aged over 60 years were at the highest risk of PJP. Age can be used to predict the risk of community-acquired pneumonia, and old age is associated with poorer PJP prognosis in both HIV-positive and HIV-negative individuals [4,41,42]. We discovered that SOT recipients with comorbid HIV, hematologic malignancies, or vasculitis were at higher PJP risk. PJP is a common pathogen in immunocompromised patients, especially those with HIV infection [2]. PJP has long been known for its high prevalence among patients with acquired immunodeficiency syndrome (AIDS) [19]. PJP is a key cause of opportunistic infection and death among patients with HIV infection [43]. Studies have reported the varying incidence of HIV-related PJP throughout the developing world [43,44,45]. Patients with hematologic malignancies are known to have higher levels of immunosuppression [46], and the incidence of PJP has been increasing in subjects with hematologic malignancy [47]. Patients with antineutrophil cytoplasmic antibody (ANCA)-associated vasculitis (AAV) were at higher risk for PJP infection [48].

We also investigate the risk difference of PJP infection between SOT recipients and CKD and HIV patients. This study found that compared with CKD patients, SOT recipients were at higher risk for PJP infection, while compared with HIV patients, SOT recipients were at lower risk for PJP infection. Immune dysfunction in CKD patients occurred independent of the underlying disease and manifested early in the course of renal insufficiency [49]. The strongest relationship was observed between incident CKD and immunosuppression for the relative duration of severe immunosuppression [50]. Subjects with CKD have the fact that they have multiple impairments of both the innate and adaptive immune systems in common [51].

The present study has several strengths. First, it used a population-based design. Patients were selected from the total population of Taiwan; thus, the large sample was representative and resulted in high statistical precision. The combination of the NHIRD with multiple data sources offered a powerful research tool. The population-based design may have also minimized selection bias, which is common in observational studies. Second, we investigated the risk of PJP among organ transplant recipients at various time points: 6 months, 1 year, 2 years, and 3 years after transplantation.

This study has a few limitations. First, the study used a claims-based dataset. In the claims-based dataset, information such as clinical manifestations, personal history, and results of laboratory tests, pathologic examinations, and imaging was not available. Therefore, PJP was identified on the basis of discharge diagnoses and not on any reports of related examinations. However, we believe that the discharge diagnoses were reliable because nearly all PJP infections resulted in hospitalization and the discharge diagnoses were coded in accordance with the results of related examinations. Second, the severity of PJP could not be precisely determined from the ICD-9-CM and ICD-10-CM codes; thus, a severity-based subgroup analysis was not feasible. Finally, we could not analyze information such as patients’ lymphocyte, CD4+ T cell, or CD8+ T cell count; these levels may have an additional positive prognostic value for PJP [52,53].

## 5. Conclusions

PJP may develop at any stage after transplantation; 6 months, 1 year, 2 years, and 3 years after transplantation, SOT recipients were at increased risk of developing PJP, with the highest risk being from more than 1 year after the SOT. In addition, patients with HIV, hematologic malignancies, or vasculitis were discovered to be at higher risk of PJP. Compared with CKD patients, SOT recipients were at a higher risk for PJP infection, while compared with HIV patients, SOT recipients were at a lower risk for PJP infection. Finally, patients aged 41–50 and above 60 years were at higher PJP risk.

## Figures and Tables

**Table 1:** Baseline characteristics of solid organ transplant recipients after matching.

Variables	Total		General Patients		SOT Recipients ^2^		*p*-Value
	N	%	N	%	N	%	
Total	52,650	100.00	42,120	100.00	10,530	100.00	
Sex ^1^							0.943
Female	20,074	38.13	16,056	38.12	4018	38.16	
Male	32,576	61.87	26,064	61.88	6512	61.84	
Age (year) ^1^							1.000
≤40	13,334	25.33	10,664	25.32	2670	25.36	
41–50	13,511	25.66	10,809	25.66	2702	25.66	
51–60	17,759	33.73	14,211	33.74	3548	33.69	
≥61	8046	15.28	6436	15.28	1610	15.29	
Mean ± SD	48.74 ± 15.11		49.07 ± 15.29		47.40 ± 14.31		
Insured salary (NTD) ^1^							0.991
≤21,000	23,655	44.93	18,919	44.92	4736	44.98	
21,001–33,000	13,500	25.64	10,805	25.65	2695	25.59	
≥33,001	15,495	29.43	12,396	29.43	3099	29.43	
Urbanization ^1^							1.000
Level 1	14,742	28.00	11,792	28.00	2950	28.02	
Level 2	17,186	32.64	13,752	32.65	3434	32.61	
Level 3	9047	17.18	7236	17.18	1811	17.20	
Level 4	7196	13.67	5755	13.66	1441	13.68	
Level 5	852	1.62	681	1.62	171	1.62	
Level 6	1748	3.32	1400	3.32	348	3.30	
Level 7	1879	3.57	1504	3.57	375	3.56	
CCI score ^1,2^							1.000
0	2160	4.10	1728	4.10	432	4.10	
1	2120	4.03	1696	4.03	424	4.03	
2	11110	21.10	8888	21.10	2222	21.10	
≥3	37,260	70.77	29,808	70.77	7452	70.77	
With comorbidities							
HTN ^2^	16,514	31.37	11,546	27.41	4968	47.18	<0.001
HPL ^2^	9831	18.67	8210	19.49	1621	15.39	<0.001
Hepatitis C	1993	3.79	773	1.84	1220	11.59	<0.001
CKD ^2^	7119	13.52	1788	4.25	5331	50.63	<0.001
Dialysis	6170	11.72	1066	2.53	5104	48.47	<0.001
COPD ^2^	4004	7.60	3526	8.37	478	4.54	<0.001
RA ^2^	696	1.32	621	1.47	75	0.71	<0.001
IBD ^2^	210	0.40	165	0.39	45	0.43	0.604
SLE ^2^	780	1.48	494	1.17	286	2.72	<0.001
Psoriasis	219	0.42	166	0.39	53	0.50	0.119
Sjogren syndrome	325	0.62	231	0.55	94	0.89	<0.001
Hematological malignancies	584	1.11	482	1.14	102	0.97	0.124
Vasculitis	11	0.02	6	0.01	5	0.05	0.035
TMP–SMX ^2^							<0.001
No	46,060	87.48	41,148	97.69	4912	46.65	
Yes	6590	12.52	972	2.31	5618	53.35	
Corticosteroid							<0.001
No	42,377	80.49	39,139	92.92	3238	30.75	
Yes	10,273	19.51	2981	7.08	7292	69.25	

^1^ Variables for propensity score matching. ^2^ Abbreviations: SOT, solid organ transplant recipients; CCI, Charlson comorbidity index; HTN, hypertension; HPL, hyperlipidemia; CKD, chronic kidney disease; COPD, chronic obstructive pulmonary disease; RA, rheumatoid arthritis; IBD, inflammatory bowel disease; SLE, systematic lupus erythematosus; TMP–SMX, trimethoprim–sulfamethoxazole. Note. Human immunodeficiency virus was included in the analysis, but one of the cells is less than three. According to the regulations of the Health and Welfare Data Science Center Ministry of Health and Welfare, Taiwan, the number cannot be present if one of the cells is less than three.

**Table 2:** Three-year follow-up of incidence *Pneumocystis jirovecii* pneumonia in solid organ transplant recipients.

Variables	*Pneumocystis jirovecii* Pneumonia									
	Adjusted Model 1					Adjusted Model 2				
	OR	95% CI			*p*-Value	OR	95% CI			*p*-Value
Patients										
General patients (ref.)	1					1				
SOT recipients ^1^	17.18	8.80	-	33.53	<0.001	-		-		-
Kidney transplant	-		-		-	15.03	6.37	-	35.44	<0.001
Liver transplant	-		-		-	16.82	8.11	-	34.89	<0.001
Lung transplant	-		-		-	62.33	16.95	-	229.22	<0.001
Sex										
Female (ref.)	1					1				
Male	1.04	0.73	-	1.48	0.850	1.03	0.73	-	1.48	0.854
Age (year)										
≤40 (ref.)	1					1				
41–50	1.75	1.09	-	2.81	0.021	1.71	1.06	-	2.74	0.028
51–60	1.24	0.74	-	2.05	0.413	1.21	0.73	-	2.02	0.454
≥61	2.01	1.14	-	3.53	0.016	1.99	1.13	-	3.50	0.017
Insured salary (NTD)										
≤21,000 (ref.)	1					1				
21,001–33,000	1.42	0.95	-	2.11	0.086	1.42	0.95	-	2.11	0.087
≥33,001	1.02	0.67	-	1.55	0.919	1.04	0.68	-	1.58	0.855
Urbanization										
Level 1 (ref.)	1					1				
Level 2	0.94	0.60	-	1.46	0.772	0.93	0.59	-	1.45	0.739
Level 3	1.54	0.96	-	2.47	0.076	1.54	0.96	-	2.47	0.075
Level 4	1.22	0.71	-	2.09	0.468	1.22	0.71	-	2.08	0.473
Level 5	1.40	0.42	-	4.69	0.585	1.41	0.42	-	4.71	0.574
Level 6	0.69	0.21	-	2.25	0.533	0.67	0.20	-	2.21	0.513
Level 7	0.58	0.18	-	1.91	0.368	0.59	0.18	-	1.93	0.380
CCI score ^1^										
0 (ref.)	1					1				
1	1.93	0.47	-	7.84	0.359	1.75	0.42	-	7.41	0.445
2	1.06	0.30	-	3.71	0.930	1.08	0.30	-	3.94	0.907
≥3	0.86	0.25	-	2.91	0.808	0.89	0.25	-	3.21	0.863
Comorbidities (yes vs. no)										
HTN ^1^	1.32	0.89	-	1.96	0.173	1.33	0.89	-	1.98	0.168
HPL ^1^	1.17	0.77	-	1.79	0.464	1.18	0.77	-	1.81	0.438
Hepatitis C	1.18	0.64	-	2.19	0.598	1.20	0.64	-	2.25	0.569
HIV ^1^	59.08	20.30	-	171.92	<0.001	58.24	19.85	-	170.87	<0.001
CKD ^1^	1.41	0.72	-	2.76	0.312	1.49	0.71	-	3.13	0.293
Dialysis	1.51	0.81	-	2.80	0.192	1.65	0.81	-	3.35	0.170
COPD ^1^	1.76	0.95	-	3.24	0.071	1.54	0.81	-	2.91	0.188
RA ^1^	0.81	0.11	-	5.93	0.833	0.82	0.11	-	5.99	0.842
SLE ^1^	0.42	0.06	-	3.09	0.395	0.42	0.06	-	3.08	0.393
Sjogren syndrome	6.33	0.64	-	62.84	0.115	6.32	0.64	-	62.70	0.115
Hematological malignancies	11.94	5.36	-	26.61	<0.001	11.26	4.67	-	27.17	<0.001
Vasculitis	21.72	2.41	-	195.81	0.006	17.50	1.85	-	165.69	0.013
TMP–SMX ^1^ (yes vs. no)	1.17	0.79	-	1.74	0.440	1.14	0.77	-	1.70	0.516
Corticosteroid (yes vs. no)	1.45	0.92	-	2.27	0.107	1.44	0.92	-	2.26	0.112

^1^ Abbreviations: SOT, solid organ transplant recipients; CCI, Charlson comorbidity index; HTN, hypertension; HPL, hyperlipidemia; HIV, human immunodeficiency virus; CKD, chronic kidney disease; COPD, chronic obstructive pulmonary disease; RA, rheumatoid arthritis; SLE, systematic lupus erythematosus; TMP–SMX, trimethoprim–sulfamethoxazole.

**Table 3:** Sensitivity analysis of incident *Pneumocystis jirovecii* pneumonia in the different observed periods.

Variables	*Pneumocystis jirovecii* Pneumonia		
	Adjusted OR ^1^	95% CI	*p*-Value
Compared with General patients			
Six-months follow-up	22.64	7.53–68.11	<0.001
One-year follow-up	26.19	9.89–69.37	<0.001
Two-year follow-up	23.06	10.23–51.97	<0.001
Compared with CKD patients ^2^			
Six-months follow-up	17.85	6.53–48.81	<0.001
One-year follow-up	13.05	6.18–27.56	<0.001
Two-year follow-up	9.85	5.25–18.48	<0.001
Three-year follow-up	10.19	5.68–18.27	<0.001
Compared with HIV patients ^2^			
Six-months follow-up	0.57	0.56–0.57	<0.001
One-year follow-up	0.28	0.27–0.28	<0.001
Two-year follow-up	0.24	0.23–0.24	<0.001
Three-year follow-up	0.23	0.16–0.33	<0.001

^1^ Adjustment of all variables as Table 3. ^2^ Abbreviations: CKD, chronic kidney disease; HIV, human immunodeficiency virus.

## Data Availability

The National Health Insurance Database used to support the findings of this study was provided by the Health and Welfare Data Science Center, Ministry of Health and Welfare (HWDC, MOHW) under license and so cannot be made freely available. Requests for access to these data should be made to HWDC (https://dep.mohw.gov.tw/dos/cp-5119-59201-113.html, accessed on 20 October 2022).

## Supplementary Materials

The following supporting information can be downloaded at: https://www.mdpi.com/article/10.3390/jof9010023/s1, Table S1: The incident of *Pneumocystis jirovecii* pneumonia in solid organ transplant recipients.
