# Supplementary material for: Risk of Pneumocystis jirovecii Pneumonia among Solid Organ Transplant Recipients: A Population-Based Study
Source: J Fungi (Basel). 2022 Dec 22;9(1):23. doi: 10.3390/jof9010023 (PMC9866281; doi:10.3390/jof9010023)
Supplement: Supplementary file 1 [file jof-09-00023-s001.zip › jof-2037923-supplementary.pdf]

Table S1. The incident of *Pneumocystis jirovecii* pneumonia in solid organ transplant recipients.

Variables	*Pneumocystis jirovecii* pneumonia				p-value
	No		Yes		
	N	%	N	%	
Total	52,507	99.73	143	0.27	
Patients					<0.001
General patients	42,101	99.95	19	0.05	
SOT recipients [1]	10,406	98.82	124	1.18	
Kidney transplant	6,090	99.56	89	1.44	
Liver transplant	4,250	99.28	31	0.72	
Lung transplant	66	94.29	4	5.71	
Sex					0.934
Female	20,019	99.73	55	0.27	
Male	32,488	99.73	88	0.27	
Age (year)					0.107
≤40	13,303	99.77	31	0.23	
41-50	13,465	99.66	46	0.34	
51-60	17,720	99.78	39	0.22	
≥61	8,019	99.66	27	0.34	
Mean ± SD	48.74 ± 15.11		48.21 ± 14.38		
Insured salary (NTD)					0.279
≤21,000	23,596	99.75	59	0.25	
21,001-33,000	13,455	99.67	45	0.33	
≥33,001	15,456	99.75	39	0.25	
Urbanization					0.455
Level 1	14,704	99.74	38	0.26	
Level 2	17,145	99.76	41	0.24	
Level 3	9,014	99.64	33	0.36	
Level 4	7,174	99.69	22	0.31	
Level 5	849	99.65	3	0.35	
Level 6	1,745	99.83	3	0.17	
Level 7	1,876	99.84	3	0.16	
CCI score [1]					0.590
0	2,157	99.86	3	0.14	
1	2,114	99.72	6	0.28	
2	11,076	99.69	34	0.31	
≥3	37,160	99.73	100	0.27	
With comorbidities					
HTN [1]	16,433	99.51	81	0.49	<0.001
HPL [1]	9,801	99.69	30	0.31	0.478
Hepatitis C	1,980	99.35	13	0.65	<0.001
HIV [1]	392	98.74	5	1.26	<0.001
CKD [1]	7,037	98.85	82	1.15	<0.001
Dialysis	6,091	98.72	79	1.28	<0.001
COPD [1]	3,992	99.70	12	0.30	0.722
IBD [1]	210	100.00	-	-	-
SLE [1]	776	99.49	4	0.51	0.192
Psoriasis	219	100.00	-	-	-
Sjogren syndrome	322	99.08	3	0.92	0.024
Hematological malignancies	576	98.63	8	1.37	<0.001

TMP-SMX [1]					<0.001
No	45,995	99.86	65	0.14	
Yes	6,512	98.82	78	1.18	
Corticosteroid					<0.001
No	42,333	99.90	44	0.10	
Yes	10,174	99.04	99	0.96	

[1] Abbreviations: SOT, solid organ transplant recipients; CCI, Charlson comorbidity index; HTN, hypertension; HPL, hyperlipidemia; HIV, human immunodeficiency virus; CKD, chronic kidney disease; COPD, chronic obstructive pulmonary disease; inflammatory bowel disease; SLE, systematic lupus erythematosus; TMP-SMX, trimethoprim-sulfamethoxazole.

Note: Rheumatoid arthritis and vasculitis had included in the analysis, but one of the cells is less than three. According to the regulations of the Health and Welfare Data Science Center Ministry of Health and Welfare Taiwan, the number can not be present if one of the cells is less than three.
